# Remotely sensing potential climate change tipping points across scales

**DOI:** 10.1038/s41467-023-44609-w

**Published:** 2024-01-06

**Authors:** Timothy M. Lenton, Jesse F. Abrams, Annett Bartsch, Sebastian Bathiany, Chris A. Boulton, Joshua E. Buxton, Alessandra Conversi, Andrew M. Cunliffe, Sophie Hebden, Thomas Lavergne, Benjamin Poulter, Andrew Shepherd, Taylor Smith, Didier Swingedouw, Ricarda Winkelmann, Niklas Boers

**Affiliations:** 1 Global Systems Institute, University of Exeter, Exeter, UK; 2 b.geos GmbH, Industriestrasse 1A, 2100 Korneuburg, Austria; 3 Austrian Polar Research Institute, Vienna, Austria; 4 Earth System Modelling, School of Engineering & Design, Technical University of Munich, Munich, Germany; 5 Potsdam Institute for Climate Impact Research, Potsdam, Germany; 6 National Research Council of Italy, ISMAR-Lerici, Forte Santa Teresa, Loc. Pozzuolo, 19032 Lerici (SP), Italy; 7 Future Earth Secretariat, Stockholm, Sweden; 8 European Space Agency, ECSAT, Harwell, Oxfordshire, UK; 9 Norwegian Meteorological Institute, Oslo, Norway; 10 NASA Goddard Space Flight Centre, Greenbelt, MD 20771 USA; 11 Department of Geography and Environmental Sciences, Northumbria University, Newcastle, UK; 12 Institute of Geosciences, University of Potsdam, Potsdam, Germany; 13 University of Bordeaux, CNRS, Bordeaux INP, EPOC, UMR 5805, 33600 Pessac, France

**Keywords:** Climate-change impacts, Projection and prediction

## Abstract

Potential climate tipping points pose a growing risk for societies, and policy is calling for improved anticipation of them. Satellite remote sensing can play a unique role in identifying and anticipating tipping phenomena across scales. Where satellite records are too short for temporal early warning of tipping points, complementary spatial indicators can leverage the exceptional spatial-temporal coverage of remotely sensed data to detect changing resilience of vulnerable systems. Combining Earth observation with Earth system models can improve process-based understanding of tipping points, their interactions, and potential tipping cascades. Such fine-resolution sensing can support climate tipping point risk management across scales.

## Abstract (web summary)

Climate change could drive critical parts of the Earth system past tipping points, causing large-scale, abrupt and/or irreversible changes that harm societies. Here, the authors suggest that satellite remote sensing can play a unique role in helping manage these profound risks, by providing improved early warning of tipping points across scales.

## Introduction

Climate change could drive some critical parts of the Earth system towards tipping points—triggering a ‘tipping event’ of abrupt and/or irreversible change into a qualitatively different state, self-propelled by strong amplifying feedback^1,2^. Crossing tipping points—triggering ‘regime shifts’^3^ or ‘critical transitions’^4^—may occur in systems across a range of spatial scales, from local ecosystems to sub-continental ‘tipping elements’^1,2^. Here, we refer to these collectively as *tipping systems*. The resulting magnitude, abruptness, and/or irreversibility of changes in system function may be particularly challenging for human societies and other species to adapt to, worsening the risks that climate change poses. Passing tipping points can feedback to climate change by e.g., triggering carbon release^5^, reducing surface albedo^6^, or altering ocean heat uptake^7^. Tipping one system can alter the likelihood of tipping another, with a currently poorly quantified risk that tipping can cascade across systems^3,8,9^ (meaning here that tipping one system makes tipping of another more likely^10^).

For all these reasons, an improved observational and modelling framework to sense where and when climate tipping points can be triggered, and how tipping systems interact, could have considerable societal value. Remote sensing data can make a unique contribution because of its global coverage at fine temporal and spatial resolution. It has played an increasingly important role in tipping point science. Early identification of tipping elements in the Earth’s climate system^1^ drew on remotely sensed evidence of accelerating loss of Arctic sea ice^11^, Antarctic Peninsula ice shelves^12^, and the Greenland^13,14^ and Antarctic^13,15^ ice sheets. Subsequently, remote sensing has provided key evidence on the location and proximity of tipping points in the polar ice sheets^16,17^, overturning classical assumptions on the pace of their response to climate change, with measurements of ice speedup^18^, thinning^19^, and grounding line retreat^16,20^, proving critical to identifying destabilisation of the West Antarctic ice sheet^21^ (WAIS). Satellite data has also been used to detect new candidate tipping elements including a strong shift in cloud feedbacks^22^, to reveal alternative stable states of boreal^23,24^ and tropical^25–27^ vegetation, and to track how vegetation resilience varies over space and time^28,29^.

Resilience is the ability of a system to recover from perturbations, which can be measured as the recovery rate. Resilience declines when approaching a tipping point^30^ providing potential early warning signals (EWS) due to critical slowing down^4^ (CSD) of system dynamics. However, resilience can also be lost in the absence of a tipping point^31^. Hence it is essential to independently identify tipping systems, e.g., using theory and evidence of alternative stable states and/or abrupt shifts in the past^32^, in spatial data, or in model simulations. Existing work^30^ proposed a resilience monitoring system for terrestrial ecosystems, irrespective of tipping, whereas here we focus on tipping systems throughout the Earth system. Previously identified tipping systems^1,2,32^ include the Greenland ice sheet^33^ (GrIS), the Atlantic Meridional Overturning Circulation^34,35^ (AMOC), and the Amazon rainforest^25^. Recently, empirical evidence of resilience loss has been detected in all three^29,36,37^, which for the Amazon was based on remotely sensed vegetation optical depth^38^ (VOD). In fast-responding tipping systems, there is a clear opportunity to leverage remote sensing data to look more widely for resilience changes. For slower-responding tipping systems, the relatively short satellite era of ~50 years is insufficient^39^. However, space-for-time substitution^25,26,40^ and spatial stability indicators^41^ can leverage the fine spatial resolution of satellite records to help forewarn of approaching tipping points. Combining Earth observations and models can improve predictions of, e.g., abrupt droughts to avert food security crises^42,43^, or abrupt loss of ecosystem function and services to inform regional policy-making and land-use planning^44^.

Here we start by highlighting policy needs for improved and sustained information on climate change tipping points and remote sensing requirements to help address those needs. Then we delve deeper into how remote sensing can help identify potential tipping points, improve resilience monitoring and early warning of tipping events, and assess the potential for tipping systems to interact and possibly cascade. In the outlook, we suggest potential ways forward and future research avenues.

## Policy needs

**Fig. 1:**
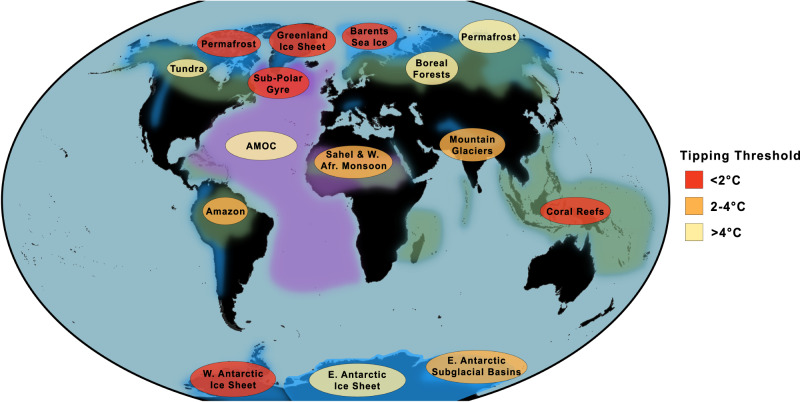
Climate tipping elements and their sensitivity to global warming based on a recent assessment^2^. Tipping elements are categorised as cryosphere (blue), biosphere (green), or circulation (purple). Colours of labels denote temperature thresholds categorised into three levels of global warming above pre-industrial (key on the right), with darker red indicating lower temperature thresholds (greater urgency). Permafrost appears twice as some parts are prone to abrupt thaw (at lower temperatures) and some (organic-rich Yedoma) to self-propelling collapse (at higher temperatures).

There are strong societal and policy drivers for improved information on potential climate tipping points because abrupt and/or irreversible, large-scale changes pose considerable risks. The risk of crossing a West Antarctic ice sheet tipping point^45^ has been recognised since the 1970s, and the IPCC’s ‘reasons for concern’ have included ‘large-scale discontinuities in the climate system’ since 2001. Over successive IPCC Reports their likelihood has been repeatedly revised upwards, such that there are now reasons for concern at present levels of global warming^46^. Figure 1 summarises currently identified climate tipping elements and their estimated sensitivity to global warming^2^, indicating that several major systems are at risk of being tipped below 2 °C. Considerable uncertainties remain and remote sensing data can help constrain them by, e.g., comparing model results to empirical evidence including identifying emergent constraints^47^, and estimating proximity to tipping points using CSD applied to remotely sensed data. Overall, interactions between tipping elements, including feedback to global temperature, are assessed to further increase the likelihood of tipping events^9,48^, although some specific interactions may decrease it^48^. The desire to avoid crossing climate tipping points has already informed mitigation policy targets including the 2015 Paris Agreement to limit warming to “well below 2 °C” and subsequent ‘net zero’ emissions pledges^49^. In our view, the risk of tipping was previously underestimated^2,46^, and this gives a compelling reason to strengthen such mitigation pledges and action to meet them.

At national-regional scales, climate tipping points could have severe impacts, such as stronger and more frequent extreme events, accelerated sea-level rise, and fundamental changes in climate variability^50^. These impacts are often distinct in their pattern and/or magnitude from those expected due to global warming alone, thus posing distinct adaptation challenges. Whether or not tipping points can be avoided by stronger mitigation policy, improved information on where and when they could occur can help guide stronger and more targeted adaptation policy. This can be aimed at reducing impacts of tipping points, exposure, and/or vulnerability to those impacts, and therefore risk^51^. Where biosphere tipping systems are (at least partly) within a national jurisdiction (e.g., boreal forests, Amazon rainforest, tropical coral reefs), remotely sensed information on an approaching tipping point could help inform national efforts to increase ecosystem resilience^30^. At local scales, the risk of climate change triggering tipping events, e.g., in ecosystems or glaciers, is a challenge for regional policy and management, which again can benefit from improved risk assessment and resilience monitoring^30^.

## Remote sensing targets and requirements

**Fig. 2:**
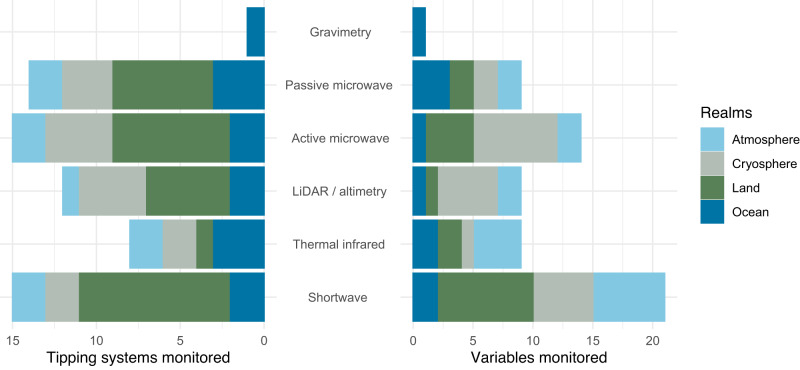
The capacity of different remote sensing methods to monitor tipping systems and pertinent variables. Summarises information in Table 1 (grouping ocean circulation and ocean biosphere together as the ‘ocean’ realm).

Given these needs, how can remote sensing of tipping systems help support policy-making and environmental management across scales? Table 1 summarises the tipping systems discussed herein, their key properties, the current utility of remote sensing for probing tipping processes, pertinent variables sensed, and methods of remotely sensing them. Figure 2 summarises the capacity of different remote sensing methods to monitor tipping systems and pertinent variables in different domains. Scientific targets for remote sensing of tipping systems include: monitoring relevant feedback processes to improve process understanding^52^; detecting alternative stable states and associated abrupt changes^53^; establishing links from alternative states and their stability to climate variables^25,26,28^; observing system dynamics over time including changes in stability or resilience, and associated early warning signals on regional^16,29^ and global^54^ scales, and; calibrating, constraining and evaluating models of tipping systems to improve predictions^17,22^.

**Table 1:** Tipping systems, their properties, and means of remotely sensing them

Realm	Tipping properties					Remote sensing							
	Tipping system	Type of tipping	Time-scale of event (yr)	Revers-iblity over 100yr?^a^	Ref(s)	Current utility^b^ in probing tipping processes	Properties sensed	Method(s) of sensing					
								Vis-Shortwave^c^	Thermal infrared	LiDAR / altimetry^d^	Active microwave	Passive microwave	Gravimetry
Ocean circulation	Atlantic Overturning (AMOC)	Macro	~10–100	No	^58^	Low	Sea surface temp. Sea surface salinity Sea level Major currents		●	●	●	● ●	●
	Sub-Polar Gyre (SPG)	Macro	~10	No	^58^	Low							
Ocean biosphere	Pelagic ecosystems	Clustered	~10	Maybe	^150^,^151^	Mid	Ocean colour Sea surface temp. Sea surface pH^e^	● ●	● ●			● ●	
	Coral reefs	Clustered	~10–100	Maybe	^152^,^153^	Mid	Habitat Sea surface temp. Wave height	●	●	●	●	●	
Terrestrial biosphere	Amazon forest	Propagating	~10	No?	^29^,^154^,^155^	High	Productivity indices Land cover types Vegetation structure	● ● ●		●	● ●	●	
	Boreal forest	Clustered	~10	No?	^23^,^24^	High							
	Forests (local)	Impact	~1–10	Yes?	^94^	Mid							
	Savanna	Clustered	~10	Yes	^25,26,113,114^	High							
	Patterned veg.	Impact	~10	Yes	^68^	Mid							
	Tidal marshes	Impact	~10	Yes	^156^	Mid	Productivity indices Water indices	● ●					
	Lake ecosystems	Impact	~1–10	Yes	^157^,^158^	Mid							
	Peatlands	Impact	~10–100	No	^159^,^160^	Mid	Water table depth Burned area	● ●			●		
	Permafrost soils	Clustered	~10–100	No	^58^,^161^	Low	Soil properties^f^ GHG concentration	● ●	● ●		●	●	
Atmospheric circulation	Monsoons (S. America, W. Africa, S. Asia)	Macro	~1–10	Yes	^42^,^162^	Mid	Precipitation Land cover change Aerosols Ozone concentration	● ● ● ●	● ●	● ●	● ●	●	
	Blocking events & heatwaves	Impact	~0.01	Yes	^42^	Mid	Land surface temp. Cloud properties	●	● ●			●	
Cryosphere	Sea ice	Impact	~1–10	Yes	^163^–^165^	High	Area and thickness			●	●	●	
	Ice shelves	Macro	<1	No	^163^–^165^	Low	Area and thickness Surface melt		●	●	● ●	●	
	Ice sheets	Macro	~100–10^4^	No	^163^–^165^	Low	Velocity Surface elevation Grounding line Surface melt	●	●	● ●	● ● ●	●	
	Mountain glaciers	Impact	~1–10	Yes	^165^	Mid	Extent Snowline Surface elevation Velocity	● ● ● ●		● ●	● ● ●		

^a^ Reversibility is considered on the timescale of a human lifespan. ^b^ Utility scale: high = key approach; mid = complementary addition to what is available from in situ data; low = offers monitoring of a process influencing tipping dynamics but insufficient to fully monitor the state of the tipping system. ^c^ Includes ultraviolet, visible to short-wavelength infra-red (VSWIR), multi-spectral and hyper-spectral methods. ^d^ Includes optical and radar. ^e^ Inferred from SST, SSS, chlorophyll. ^f^ Inferred from land cover types, land surface temperature and snow dynamics.

Building on previous work^42^, we propose a minimum set of ideal criteria for remotely sensed datasets to be useful in tipping point applications: (1) Salient variables correlated with key processes underlying tipping dynamics and their possible interactions. (2) Accurate, analysis-ready data. (3) Spatial coverage of the tipping systems of interest. (4) Spatial resolution sufficient to resolve key feedbacks involved in tipping dynamics. (5) Temporal resolution sufficient to resolve timescales of tipping or recovery (Table 1). (6) Temporal duration sufficient to estimate system resilience, and ideally to detect changes in forcing and resilience. (7) Low data latency to support timely detection and/or early warning of tipping points.

Box 1 expands on current remote sensing capabilities and limitations in relation to these criteria.

Box 1 Remote sensing capabilities and limitations in relation to tipping point criteria Current remote sensing has capabilities and limitations in relation to a minimum set of ideal criteria for tipping point applications^42^:

**Salient variables correlated with tipping processes**. The increasing diversity of geophysical parameters retrievable from satellites widens capabilities, but established metrics (e.g., normalised vegetation difference index; NDVI) can be limited in their ability to probe variables prone to tipping (e.g., biomass)^30^. Consistency between remote sensing data records also needs to be more extensively studied^145^ to reliably link tipping phenomena to climate variables.

**Accurate, analysis-ready data**. Several operational services make pre-processed, calibrated, and validated datasets available to defined standards, but accuracy and coverage can still be inconsistent in space and time, biasing resilience estimates. Notably optical and thermal infrared data require masking (e.g., for cloud) and correcting for atmospheric attenuation, adding to retrieval uncertainty and making inferences of resilience less reliable^91^. This is compounded by artefacts introduced by merging and harmonising observations from multiple sensors^141^ (see Box 2).

**Spatial coverage of tipping systems**. Polar-orbiting satellites help provide global coverage, but monitoring of below-surface ground and water is limited, restricting sensing of e.g., permafrost or the AMOC. Optical (passive shortwave) measurements are limited by sunlight availability and cloud cover, seasonally restricting sensing of e.g., sea ice, ice sheets, and tropical or boreal forest. Synthetic aperture radar (SAR) data (active microwave) are illumination independent, unaffected by cloud, and can monitor many tipping systems, but have inconsistent coverage due to frequent switching of modes according to user or security interests.

**Spatial resolution is sufficient to resolve tipping dynamics**. The wide range of very fine resolution (<1 m) and fine resolution (<10 m) satellite constellation missions and sensing types facilitate space-for-time substitution, derivation of spatial stability indicators, and pattern change detection. However, lack of open access to very fine-resolution data, short collection timespans, and computational overheads of data analysis pose challenges, whilst cross-calibration and co-registration of pixels restrict suitable precision for ecological applications.

**Temporal resolution is sufficient to resolve timescales of tipping or recovery**. Regular revisits (relative to system variability) allow the detection of abrupt changes, and perturbations, and the calculation of temporal resilience indicators (e.g., CSD indicators as proxies of recovery rate). Upscaling techniques using data from geostationary missions with sub-hourly observations and exploiting SAR or passive microwave sensor data (e.g., VOD products), may help fill data gaps due to cloud cover and insufficient revisits, particularly in the tropics.

**Temporal duration is sufficient to estimate system resilience**. Continuous time series of multi-decadal length for some variables are valuable for identifying acceleration of processes (e.g., ice melt; forest dieback), detection of abrupt shifts, and analysis of changing variability and resilience for potential early warning in fast (e.g., ecological) tipping systems, but are insufficient in slow tipping systems (e.g., AMOC or Greenland ice sheet).

**Low data latency to support timely detection and early warning of tipping points**. Near-real-time data are now available for many remote sensing products enabling resilience monitoring even for very fast tipping systems^42,43^. However, latencies are still limited by revisit frequency and the time of overpass, which may bias observations, e.g., to miss peaks in fire coverage, plant water stress, or meteorological extremes.

## Remote sensing opportunities

Having established these criteria, we now identify and elaborate key opportunities for remote sensing to advance the understanding and detection of different tipping phenomena, of changing resilience, and of interactions between tipping systems.

### Detecting different tipping phenomena

Remote sensing can advance the detection of different types of tipping phenomena across scales (Table 1), which pose different remote sensing challenges and opportunities.

#### Crossing scales

The most impactful tipping points can be divided into four categories: Impacts can result from tipping inherently large-scale tipping elements (macro tipping), or from localised tipping points that interact to cause larger-scale change (propagating tipping) or are crossed coherently across a large area (clustered tipping) or initiate significant consequences in social systems (societal impact tipping). Large-scale tipping elements have generally been identified^1,2^ from the ‘top down’, e.g., from conceptual models, understanding of key feedbacks, and/or paleoclimate records of large-scale past abrupt changes^32^. Meanwhile, localised tipping systems have principally been identified from the ‘bottom up’ by direct observations^55,56^. Remote sensing can simultaneously identify and monitor tipping systems, phenomena, and their interactions across scales.

#### Macro tipping

For tipping elements involving atmospheric circulation (e.g., monsoons), ocean circulation (e.g., AMOC, sub-polar gyre; SPG), or ice sheets (e.g., GrIS, WAIS), the crucial reinforcing feedback mechanisms that can propel tipping operate across large spatial scales. The global coverage of remote sensing uniquely enables comprehensive observation at the large scale of those feedbacks. Even where a system is only partially observable, remotely sensed data can reveal underlying (in)stability. For example, remote sensing provides unique opportunities to identify large-scale expressions of SPG and AMOC circulation strength and associated stability changes in fingerprint patterns in sea surface temperature (SST), salinity (SSS), or height (SSH) in specific areas (e.g., Labrador Sea and Nordic Seas) where models suggest a link between these observable fingerprints and proximity to tipping points^57^. Remote sensing of deep ocean pressure from gravity field changes also reveals below-surface characteristics relating to AMOC strength^58^. Remote sensing of fine-scale properties across large areas can be used to recalibrate process-based models to improve assessments of large-scale tipping potential. For example, assimilating remotely sensed rainfall data can improve short-term monsoon forecasts^59^. Correcting modelled cloud ice particle content has revealed the possibility of much higher long-term climate sensitivity^22^. Furthermore, progress is being made assimilating remotely sensed ice-surface velocity and elevation changes into high-resolution models of Antarctica^60^.

#### Propagating tipping

Large-scale tipping elements can in some cases (e.g., WAIS, Amazon rainforest), be considered as networks of smaller, coupled components within which propagating tipping may occur due to causal interactions. In rare cases, tipping of the most sensitive components may ultimately destabilise the rest in a ‘domino cascade’^10^. The comprehensive coverage of remote sensing at high spatial and temporal resolution is uniquely able to detect propagating tipping by monitoring pertinent localised phenomena and larger-scale responses. For example, several localised tipping points of the Pine Island glacier are theoretically able to destabilise the Amundsen basin^61^, in turn risking the whole West Antarctic ice sheet^62^. Satellite-based radar altimetry has detected both the localised grounding line retreat of glaciers^16^ and confirmed that ice dynamical imbalance has spread to one-quarter of the WAIS since the 1990s^19^. Another example is the Amazon rainforest, where if dieback starts in the northeast it may propagate southwest—along the prevailing low-level wind and moisture transport direction—through the reduction of rainfall recycling by the forest^63^. Alternatively, dieback or deforestation starting in the drier southeast may propagate through drying the local climate and enhancing fires. Continuous satellite-based drought^42^ and fire monitoring are crucial to detect where the forest is at risk of tipping and any propagating tipping. Remote sensing can also track human activities of deforestation, land-use change^64^, and associated forest fragmentation^65^ that may trigger tipping. At ecosystem scales, remote sensing can detect propagating tipping, e.g., in the form of propagating ‘invasion fronts’ where one bi-stable ecosystem state replaces another^66^. It can also monitor potential inhibition of propagating tipping by damping feedback at larger scales, for example in patterned vegetation systems^67,68^.

#### Clustered tipping

Where spatial coupling is less strong, localised tipping may still occur in clusters near-synchronously across a large area, due to a spatially coherent climate or anthropogenic forcing reaching a common threshold, e.g., widespread coral bleaching, thermokarst, and lake formation in degrading permafrost, or synchronous forest disturbances^69^ and dieback. Remote sensing is key to detecting clustered tipping and assessing its spatial scale, for example through the application of abrupt change detection algorithms^70^—e.g., change point analysis applied to dryland ecosystems^53,71^ or general trend retrieval applied to thaw lakes across the Arctic^72^. Remote sensing across environmental gradients is also key to assessing where clustered tipping could occur, helping detect multiple attractors and thus the potential for local tipping points, using e.g., tree cover with respect to rainfall. Early studies suggested widespread multi-stability of tree cover along rainfall gradients in tropical^25,26^ and boreal^23^ regions. However, other potential causal drivers of multimodality—notably human activities—can shrink the areas of true bistability^24,27,73^. Remote sensing of the Global Climate Observing System’s Essential Climate Variables^74^ (GCOS ECV) can also provide evidence of pertinent feedbacks, e.g., localised forest-cloud feedbacks^75^ or large-scale alteration of carbon sinks^76^ (e.g., by permafrost thaw or forest dieback).

#### Societal impact tipping

Remote sensing can detect localised tipping points in the provision of ecosystem services, which can have substantial impacts on societal systems, where tipping intersects with high human population density. For example, the abrupt loss of glaciers that feed dry-season runoff can have severe impacts on agricultural irrigation downstream^77^, and agricultural systems may exhibit their own tipping points in the delivery of ecosystem services^78^. Another example is the amplification of persistent heatwaves by land surface drying and atmospheric heat storage^79^, with potentially severe impacts—e.g., Europe 2003, Russia 2010, and North America 2021. Remote sensing data are already an essential part of ensemble forecasting of atmospheric blocking events and helped detect amplifying feedbacks and resultant impacts on the biosphere^80^, including wildfires^81^, affecting air pollution and human health^82^. Remote sensing is also used for early warning of droughts and food security crises^42,43^. Impacts of heatwaves and drought can further cascade through social systems, e.g., when the 2010 drought in Russia harmed wheat production, exports were restricted, contributing to an escalating global wheat price, which is implicated in the ‘Arab Spring’^83^. However, empirical research is needed to establish what is a social tipping point to avoid misuse of the concept^84,85^.

### Resilience monitoring and tipping point early warning

**Fig. 3:**
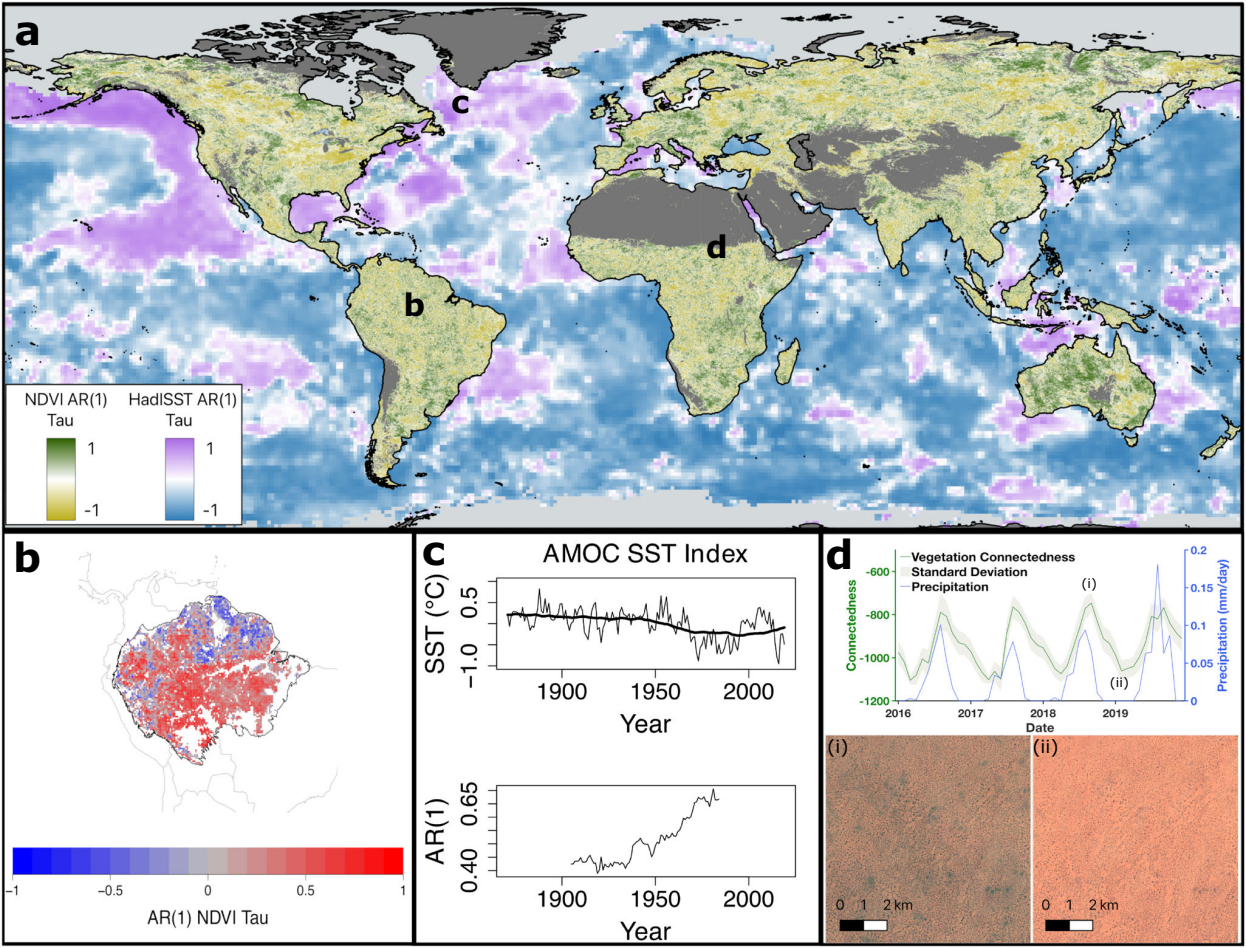
Sensing the changing resilience of tipping systems directly from observations. Examples over different time intervals using directly and remotely sensed data: **a** Trends in lag-1 temporal autocorrelation (AR(1)) of global vegetation^30^ from monthly MODIS NDVI for 2001-2020, and of global sea surface temperatures (following the approach of ref. ^166^) from monthly HadISST for 1982-2021 (which includes AVHRR data). AR(1) trends are measured with Kendall’s τ rank correlation coefficient, with darker green (vegetation) and darker purple (SST) indicative of greater loss of resilience. Light grey areas correspond to pixels with sea ice and dark grey areas to those with low NDVI ( < 0.18) values. **b** Trends in AR(1) in the Amazon rainforest (Kendall τ) from AVHRR NDVI for 2003-2016 (redrawn from ref. ^29^). **c** Changes in SST in the Sub-Polar Gyre region from HadISST for 1870-2019 (upper panel) and associated upward trend in AR(1) (lower panel) suggesting loss of resilience of the AMOC (redrawn from ref. ^37^). **d** Fluctuations in patterned vegetation connectedness and rainfall at a site in the Sahel^68^ (11°37’12”N, 27°51’36”E) from Sentinel-2 and ERA5 precipitation data for 2016–2019 (upper panel), between seasonal extremes of (i) maximum and (ii) minimum connectedness (lower panels). Connectedness is quantified from feature vectors with an ‘Offset50’ metric defined in ref. ^68^. By measuring the decay rate of connectedness between maxima and minima, averaged over years, and compared across sites, the resilience of these dryland systems is found to decline with rainfall^68^.

Relatively long remote sensing records, and new techniques to harmonise continuous observations over time for Essential Climate and Biodiversity Variables^86,87^, offer new opportunities for monitoring resilience (Box 2) and providing early warning signals (EWS) of some tipping points (Fig. 3).

#### Critical slowing down

Established early warning methods hinge on the phenomenon of critical slowing down (CSD): relatively slow forcing towards a tipping point where a system’s state loses stability, causing overall negative feedback to get weaker, slowing system dynamics including the recovery rate from small perturbations—i.e., loss of resilience. Non-tipping systems may also lose resilience^31^, hence additional, independent evidence of strongly amplifying feedback and/or empirical or paleoclimate evidence of past tipping, should be used to identify tipping systems. For candidate biosphere tipping systems such as tropical rainforests, boreal forests, and possibly drylands^53^, remote sensing provides unique opportunities to monitor resilience changes that have only begun to be exploited. Vegetation Optical Depth (VOD) data recently enabled the first global-scale empirical confirmation of CSD theory, by comparing recovery rates from perturbations with estimates based on the CSD indicators variance and lag-1 autocorrelation^54^.

#### Temporal resilience indicators

Where repeated perturbations are known to occur, changes in recovery rate can be directly monitored^54,88,89^. However, in most cases, resilience can only be inferred from changes in temporal autocorrelation (e.g., at lag-1; AR(1)) and variance^90^. These temporal EWS all require a separation of timescales: a system should be forced slower than its intrinsic response timescale for it to remain close to equilibrium. Furthermore, to detect CSD, a system must be monitored over the forcing timescale, and at a higher frequency than its response timescale. The intrinsic recovery timescales of different tipping systems range from the order of days (atmospheric circulation) or months (vegetation) to millennia (ice sheets). Climate forcing is occurring on multi-decadal to centennial timescales hence some intrinsically ‘slow’ tipping systems may not show CSD in practice. The longest ~50-year remote sensing records (i.e., Landsat) manage to capture the forcing timescale, but only the responses of relatively ‘fast’ tipping systems are monitorable with temporal EWS (those in Table 1 with a timescale of change ~10 years).

#### Resilience sensing of vegetation

Ecosystems are highly complex and only a subset are tipping systems, but they risk abrupt losses of functionality, with the potential to find CSD in remotely sensed data. The reliability of temporal resilience indicators, given measurement noise and data gaps, has been carefully assessed for NDVI and similar optical indices across major biomes^91^ and at the global scale^92^. The predicted relationships between recovery rate and autocorrelation or variance resilience indicators have also recently been confirmed for vegetated ecosystems at a global scale^54^, based on VOD and NDVI. Hence autocorrelation and variance can be used to measure vegetation resilience changes over time at high spatial resolution using remotely sensed data (Fig. 3a, b). Globally, during the last two decades, the fraction of land surface exhibiting resilience losses has increased^54^ compared to the 1990s. Focusing on tipping systems: The Amazon rainforest shows a large-scale loss of resilience^29^ over the past 20 years in VOD and NDVI (Fig. 3b), which peaked during two severe Amazon drought events in 2005 and 2010 and is greatest in drier parts of the forest and places closer to human activities (whereas during the 1990s resilience was being gained^29,54^). For boreal forests, NDVI fluctuations have a poor fit to an autoregressive model across large areas^93^, whilst VOD fluctuations^54^ suggest slow recovery rates (low resilience), with a heterogeneous pattern of resilience losses and gains across space. Smaller scale, societal impact tipping systems (Table 1) include forest regions subject to dieback, and analysis of Californian forests has shown CSD in NDVI prior to forest dieback events^94^. Opportunities for future progress depend crucially on improvements in remote sensing datasets highlighted in Box 2.

#### Application to other tipping systems

Although some ocean and cryosphere tipping elements are expected to be too slow to show temporal EWS in current Earth observation records, changes in the mean state, more localised tipping events, and some crucial feedbacks may be faster, more detectable, and informative of resilience changes. CSD has been detected in the analysis of ice-core-derived height variations of the central-western Greenland ice sheet^36^ over the last ~170 years—although contrasting patterns of mass loss acceleration in different basins indicate a complex picture^95,96^. As remote sensing records get longer, they can play a key role in comprehensively monitoring the dynamic state of the Greenland ice sheet. CSD has also been detected in proxies of AMOC strength^37^ from Atlantic SST (Fig. 3c) and SSS fluctuations observed over the last ~150 years and reconstructed over the last millennium^97^. Remote sensing can offer additional process-based monitoring of tipping processes in the Atlantic circulation. For instance, a large change in the sub-polar gyre (SPG) should be preceded^98^ by large and characteristic changes in SST and SSS, followed by changes in SSH as regional circulation changes. The same may be true for deep convection in the Nordic Seas, a key part of the AMOC. Other relatively fast tipping systems with the potential for remotely sensed temporal EWS include coral reefs^99^, monsoons, and atmospheric blocking events^100^ (Table 1). Although highly uncertain, the risks from very fast tipping in atmospheric circulation systems, including monsoons, demand continuous monitoring that remote sensing can provide^59^. Moreover, consistent increases in lag-1 autocorrelation of soil moisture have recently been found prior to drought-related changes in food security^43^, demonstrating EWS as an important potential driver of societal impact tipping.

#### Noise-induced tipping

Where the resilience of a tipping system is low and short-term variability in forcing (‘noise’) is sufficiently high, noise-induced tipping may occur without forewarning^101^. This includes cases of fast forcing of slower tipping systems (where CSD is not expected or detectable) and of increasing climate variability and extremes triggering tipping^102^. Remote sensing can help assess the statistical likelihood of noise-induced tipping^103^ through monitoring both system resilience and forcing variability (see ‘Deriving tipping probabilities’, below).

Box 2 Improving remote sensing of resilience Recent studies^29,30,54,91–94^ analysing vegetation resilience using remotely sensed data have highlighted some limitations and opportunities for improvement that are also relevant beyond the biosphere:

**Data discontinuities**. Even geostationary satellites do not continuously measure surface parameters. Temporal aggregation (e.g., MODIS vegetation data is provided as 8-day composites) can create pseudo-continuous records from discontinuous data, but cannot reliably fill long gaps e.g., due to cloud cover. Discontinuities in data records can have strong impacts on inferred system resilience and changes therein by biasing the variance and autocorrelation of a data set^91^. Novel resilience estimates that account for these discontinuities—or that do not require continuous data in the first place—are needed to take full advantage of remote sensing data.

**Uncertain data**. Satellite missions have been flown with vastly different design parameters and are often repurposed for novel applications. For example, AVHRR data has been used in several long-term studies of vegetation resilience^29,54,93^, despite being originally designed for atmospheric monitoring. The value of its relatively long sensing period (1979-) is limited by calibration problems, orbital drift, and wide sensing bands; while long-term trends in the mean provide valuable insight, changes in higher-order statistics throughout the lifespan of a sensor can propagate into resilience metrics^141^, and hence add uncertainty to any resilience analysis. Modern sensors provide better data, with the constraint of a relatively short instrument record; cross-referencing lower-quality data records with their modern improvements helps bridge this temporal gap (e.g., ESA’s Climate Change Initiative^146^).

**Merging sensors**. Composite data records combine multiple satellite missions into a single continuous record; VODCA is composed of passive microwave data from seven satellites with similar spectral properties^38^. While providing a single long-term record, this also introduces potential biases when used^29,54^ to estimate resilience. Changes in sensor fidelity and merging overlapping measurements both alter data quality through time; all else being equal, this will drive anti-correlation in AR(1) and variance^141^. This behaviour is opposite to the positively correlated increases in both metrics experienced by a system moving towards a tipping point, providing a means to distinguish their signals^141^. Nevertheless, single-sensor instrument records should be preferred for the estimation of resilience, and special attention must be paid to the construction of multi-instrument records so that they do not bias the inferred resilience of a system.

**Interpretation**. How to interpret remotely sensed resilience estimates remains a fundamental issue^30^. Most studies focus on NDVI which relates a spectral ratio to vegetation properties, most notably chlorophyll content and photosynthetic activity^30,147^. However, optical data only sample the canopy surface—particularly when vegetation is dense. Hence NDVI resilience is not whole-plant resilience^30^. VOD is influenced by plant water content and structure, which in turn can be correlated with biomass^29^, suggesting VOD resilience is closer to whole-plant resilience^30^. However, passive microwave (e.g., VOD) can also be influenced by surface water^148^, suggesting active microwave^149^ (radar) is slightly more robust for monitoring biomass resilience. In general, translating spectral properties into in situ ecosystem changes involves many simplifying assumptions, adding uncertainty to resilience assessments. A systemic effort to deduce which remotely sensed properties best reflect vegetation resilience is overdue.

### Leveraging spatial data

The spatial coverage and fine resolution of remotely sensed data offer additional underutilized opportunities for resilience sensing and potential tipping point early warning, especially where the temporal duration of data is limited.

#### Space-for-time substitution

Space-for-time substitution assumes that changes in properties along spatial environmental gradients are equivalent to the response of a system to temporal changes in the same environmental driver(s). For example, where temporal remotely sensed data is sufficient to estimate resilience indicators (e.g., AR(1)) at each location, but not to detect changes in them, looking across gradients in environmental drivers can reveal how resilience varies, e.g., how resilience of tropical forests is generally lower in regions with less mean annual precipitation^28^. It is important to account (where possible) for other factors that also vary spatially and may influence resilience, e.g., using a linear additive model^28^, recognising that data for some of these factors may not be remotely sensed, e.g., soil fertility^28^. Also, additional information should be used to determine whether declining resilience may indicate an approach to a tipping point.

#### Deriving tipping probabilities

Using space-for-time substitution, remotely sensed data can be used to derive probability density functions for vegetation states, for different climate boundary conditions, e.g., mean annual precipitation. Characterising how weather variability drives vegetation variability, the resilience and size of the basin of attraction (of a current stable state) can be inferred, and from that, probabilities of leaving that state (through noise-induced tipping). Applying this approach to remotely sensed annual tree cover fraction reveals that the most resilient parts of the Amazon rainforest are those that have experienced stronger interannual rainfall variability in their long-term past^40^. In cases where available time series show frequent transitions between alternative attractors, tipping probabilities can be estimated directly^103,104^, e.g., using paleoclimate data and lake data^103^. Hence, remote sensing data for systems that have undergone multiple abrupt shifts, such as lakes^105^, could be used to estimate tipping probabilities.

#### Spatial early warning indicators

EWS in spatially extended systems depends on the nature of spatial interactions, which remote sensing can help resolve. For macro tipping, responses are expected to be spatially homogeneous, whereas, for other tipping phenomena involving heterogeneous feedbacks and spatial interactions, these can determine the scale of tipping^106^ and where EWS are expected^107^. For example, reduced rainfall could lead to vegetation change at different times in different places, but the locations may be causally linked via moisture recycling feedbacks^107–109^. Spatial EWS can be expected as increases in spatial variance or skewness^4,110,111^ and cross-correlations^41^. The detection of spatial EWS requires that a tipping system is monitored at least at the spatial resolution over which its reinforcing feedbacks manifest spatially^112^. Fine-resolution remote sensing can enable this, as demonstrated across rainfall gradients in the tropics where spatial EWS have been found before the switch of savanna/forest vegetation types^113,114^.

#### Spatially patterned systems

In systems with regular spatial structure, such as the patterned vegetation found in drylands^68^ (Fig. 3d), spatial self-organisation (creating multiple stable patterns states at low rainfall) may enable ecosystems to evade a tipping point and associated abrupt loss of ecosystem services^67^. Plant-soil feedback can be key to spatial self-organisation and affect community assembly and resilience above and below ground^66^. Fine spatial resolution remote sensing data that are continuous in time across large areas has enabled quantification of pattern connectedness and monitoring of its temporal variation as an aboveground resilience indicator^68^ (Fig. 3d). However, to fully unravel ecosystem complexity also requires complementary approaches, including on-site field studies.

### Combining data and models

Combining remotely sensed data and Earth system models offers opportunities to improve forecasting of tipping points, which is crucial given persistent parametric and structural errors in the ability of models to predict tipping points^32^.

#### Designing remote sensing strategies

Earth system models can guide where spatially to look for temporal EWS, for example in ocean circulation^39^ or ice sheets^61^. Model simulations could also help identify which processes and where best to remotely monitor for spatial EWS of a tipping point. For example, examining simulated SSH, SST, and SSS data prior to modelled abrupt shifts in the sub-polar gyre^57,98^, incorporating known uncertainties in remote sensing, could determine which remotely sensed data are most informative for EWS and where additional monitoring could add value.

#### Emergent constraints

Emergent constraints^47^ describe a semi-empirical method whereby models identify observable targets that can constrain future predictions^115^. Emergent constraints allow observational (including remotely sensed) data to constrain the distribution of long-term projections from a large multi-model ensemble. Most focus has been on linear responses, e.g., precipitation forecasts^116^, but emergent constraints can be developed and applied to tipping responses, as has been done for SPG instability^57,98^ and Amazon dieback^117^. Theoretical progress is needed to build confidence in this relatively sensitive and empirical approach.

#### Decadal predictions

Decadal climate predictions already highlight the benefit of initialising climate models from observed states^118^, assimilating the best available observations, including from remote sensing for spatial-temporal coverage^119^. This approach greatly improves the predictability of the North Atlantic Oscillation^120^. There is a clear opportunity to apply it to tipping elements with a decadal memory component. For instance, the SPG shows abrupt changes in several CMIP5 and CMIP6 model simulations^57,98^, but it is unclear how close this tipping point is or how it is mechanistically related to the AMOC. Initialising those models with remotely sensed data could provide an improved assessment of tipping event timing, tipping system interactions, and, through large ensembles, a statistical assessment of likelihood. Decadal prediction systems are now going beyond climate models to full Earth system models^121,122^, opening further opportunities for the assimilation of remotely sensed data to improve forecasting of ‘fast’ biogeochemical tipping systems e.g., Sahel vegetation.

### Assessing tipping interactions

Remote sensing can provide critical information to improve the assessment of interactions between tipping systems, including the potential for cascades^10^, by regularly and consistently observing multiple modelled variables across space and time.

#### Current understanding

Current assessments of tipping interactions come from paleoclimate proxy data^8^, expert elicitation for a subset of tipping elements^123^, model studies of specific tipping element interactions^124^, or qualitative assessment across different scales of tipping system^3^. Idealised models have been used to assess the transient^48^ or eventual equilibrium^9^ response to the combined effects of interactions amongst a subset of tipping elements—suggesting they increase risk overall^9,48^, lowering tipping point thresholds^48^—but this is based on a dated expert elicitation^123^. Large uncertainties remain over whether particular interactions are net stabilising or destabilising^123^.

#### Detecting interactions

Remotely sensed data (Table 1) can provide crucial information to detect or validate tipping system interactions predicted by Earth system models and constrain their signs and strengths. Taking a previously identified example of a key interaction chain^46^, backed up by detailed model studies^125^: Rapid melting of the GrIS is already well-observed by altimetry and gravimetry and is predicted to increase the likelihood of crossing tipping points in the sub-polar gyre (SPG) circulation and the AMOC^124^—albeit dependent on model and resolution^126–128^. Associated changes in North Atlantic SSS, SST, and the SPG circulation should be observable through passive microwave, thermal infrared, and altimetry, respectively. Effects on AMOC strength should also become detectable in SST and SSS spatial fingerprints. Models, paleo-data^129,130^ and the observational record show that AMOC weakening shifts the intertropical convergence zone (ITCZ) southwards, affecting tropical monsoon systems, but whether this has a destabilising^9,123^ or stabilising^131,132^ effect on the Amazon rainforest is currently unclear. Remotely sensed data can help resolve this through detecting movements in the position of the ITCZ, variations in tropical Atlantic SSTs, and resulting changes in precipitation, water level, and storage over the Amazon region in outgoing longwave radiation, radar, radar altimetry, and gravimetry. Destabilisation of the Amazon rainforest is already identifiable in VOD and NDVI^29^. Amazon deforestation and/or climate change-induced dieback might trigger monsoon shifts because the South American monsoon depends critically on evapotranspiration from the rainforest^108^, which can be probed with a combination of remote sensing and models.

#### Inferring causality

Applying methods of data-driven causality detection^133^ to time series^134^ and across spatial^135^ remotely sensed data can help establish causal relationships between tipping systems and eliminate confounding factors in apparently coupled changes. Remotely sensed data provide a promising basis to recover the network of interactions between faster tipping systems, building on the successful recovery of causal connections between geophysical variables^136^, including ice cover in the Barents Sea tipping element^2^ and mid-latitude circulation^134^ and Walker circulation couplings in the equatorial Pacific^137^. Remotely sensed data could also be used to infer causal effects of climate on this network, building on success for vegetation^138,139^. For fast tipping systems that have undergone abrupt shifts, convergent cross-mapping^140^ could be attempted to establish whether a deterministic nonlinear attractor can be recovered from remotely sensed data.

## Outlook

We have highlighted the unique value that satellite remote sensing, with its global coverage, fine resolution, and increasing diversity of variables, can bring to advancing the understanding, detection and anticipation of climate change tipping points, and their interactions, across scales. Given the risk that tipping points pose this should be urgently informing both future missions and the extraction of information from existing remotely sensed data. Here we recommend key areas for advancing research to remotely sense climate change tipping points across scales.

### Sensing system

Establishing a tipping point sensing system would provide a unifying research framework to bring together the Earth system and Earth observation communities. It would combine data and models to identify and anticipate potential tipping points, demanding advances in data, methods, and analysis. This should start with a systematic scan of existing remotely sensed data to detect abrupt shifts and regions of multi-stability with the potential for future tipping, and a systematic analysis of the potential for temporal and spatial EWS in faster tipping systems, given current data or prospective missions. Models should guide what and where to monitor tipping processes and temporal and spatial EWS in remotely sensed data. Conversely, remote sensing data should be used to constrain model projections of the location and timing of tipping points under specific forcing scenarios. The resulting tighter integration of observations, models, and theory would address the urgent need for improved scientific information on tipping point risks to inform policy.

### Improving data

The veracity of a tipping point sensing system depends crucially on improving the salience, accuracy, continuity, and consistency of remotely sensed data. We recommend revising acquisition strategies and exploiting special constellations (e.g., synchronised orbits, bistatic or multi-static radar) toward smarter use of existing remotely sensed data. To make ecosystem monitoring more salient, calls for improved vegetation indices that link to key properties such as biomass (e.g., utilising active microwave). Enhancing data accuracy calls for ongoing utilisation of cloud-insensitive wavelengths (e.g., SAR) with improved consistency of coverage. Temporal resilience sensing would benefit from enhanced access to single-sensor data, and improved multi-instrument records that minimise introducing artefacts^141^ (see Box 2). Spatial resilience sensing would benefit from open access to very high-resolution spatial data and computational power to analyse it.

### Refining methods

The veracity of a tipping point sensing system also depends on refining methods of analysing remotely sensed data. Recent advances in training deep learning to detect and provide early warning of tipping points^142,143^ should be applied to remotely sensed data, including using segmentation algorithms to complement edge detection in spatial data^144^. Methods of estimating resilience based on spatial statistics should be applied to the high spatial but low temporal resolution of some existing (e.g., Landsat) and new (e.g., GEDI) data, and duly refined. New data (e.g., GEDI, Sentinel-6, EnMAP) demand resilience sensing methods that limit the impacts of data discontinuities, of merging signals from different sensors, and of low temporal resolution. The comparison of recovery rates measured after perturbation and inferred from AR(1) and variance should be extended beyond vegetation indexes^54^ to underpin the wider application of temporal EWS. Multivariate Earth observations should be used to help resolve different mechanistic explanations for observed increases in autocorrelation and variance, e.g., combining vegetation indexes (such as NDVI and VOD), rainfall statistics, and deforestation data to understand signals of changing Amazon rainforest resilience^29^.

## Conclusion

The resulting fine-resolution spatial-temporal sensing of tipping systems can support policy-making and risk management at regional, national, and international scales. It can actively help to protect numerous human lives and livelihoods that are at risk from climate change tipping points. Return on investment is also expected to be good, as the framework for Earth observation technology is largely in place and expanding rapidly with commercial partnerships with public agencies. Key opportunities lie in smarter use and combination of existing remote sensing data to detect and forewarn of tipping points across scales.

## Data Availability

All data used in Fig. 3 are freely available from the following sources: MODIS data from NASA: https://modis.gsfc.nasa.gov/data/. HadISST data from the Met Office: https://www.metoffice.gov.uk/hadobs/. AVHRR NDVI data from USGS: https://www.usgs.gov/centers/eros/science/usgs-eros-archive-avhrr-normalized-difference-vegetation-index-ndvi-composites. Sentinel-2 data from Copernicus: https://dataspace.copernicus.eu/explore-data/data-collections/sentinel-data/sentinel-2. ERA5 precipitation data from Copernicus: 10.24381/cds.adbb2d47. The AMOC SST Index can be found as ‘SST_SG_GM’ in ref. ^37^.

## References

[1] Lenton TM (2008). Tipping elements in the Earth’s climate system. Proc. Natl Acad. Sci. USA.

[2] Armstrong McKay DI (2022). Exceeding 1.5 C global warming could trigger multiple climate tipping points. Science.

[3] Rocha JC, Peterson G, Bodin Ö, Levin S (2018). Cascading regime shifts within and across scales. Science.

[4] Scheffer M (2009). Early warning signals for critical transitions. Nature.

[5] Gasser T (2018). Path-dependent reductions in CO_2_ emission budgets caused by permafrost carbon release. Nat. Geosci..

[6] Wunderling N, Willeit M, Donges JF, Winkelmann R (2020). Global warming due to loss of large ice masses and Arctic summer sea ice. Nat. Commun..

[7] Liu W, Fedorov AV, Xie S-P, Hu S (2020). Climate impacts of a weakened Atlantic Meridional Overturning Circulation in a warming climate. Sci. Adv..

[8] Brovkin V (2021). Past abrupt changes, tipping points and cascading impacts in the Earth system. Nat. Geosci..

[9] Wunderling N, Donges JF, Kurths J, Winkelmann R (2021). Interacting tipping elements increase risk of climate domino effects under global warming. Earth Syst. Dynam..

[10] Klose AK, Karle V, Winkelmann R, Donges JF (2020). Emergence of cascading dynamics in interacting tipping elements of ecology and climate. R. Soc. Open Sci..

[11] Comiso, J. C., Parkinson, C. L., Gersten, R. & Stock, L. Accelerated decline in the Arctic sea ice cover. *Geophys. Res. Lett.* **35**, L01703 (2008).

[12] Cook AJ, Vaughan DG (2010). Overview of areal changes of the ice shelves on the Antarctic Peninsula over the past 50 years. Cryosphere.

[13] Velicogna I, Wahr J (2006). Measurements of time-variable gravity show mass loss in Antarctica. Science.

[14] Rignot E, Kanagaratnam P (2006). Changes in the velocity structure of the Greenland ice sheet. Science.

[15] Thomas R (2004). Accelerated sea-level rise from West Antarctica. Science.

[16] Rignot E, Mouginot J, Morlighem M, Seroussi H, Scheuchl B (2014). Widespread, rapid grounding line retreat of Pine Island, Thwaites, Smith, and Kohler glaciers, West Antarctica, from 1992 to 2011. Geophys. Res. Lett..

[17] Joughin I, Smith BE, Medley B (2014). Marine ice sheet collapse potentially under way for the Thwaites Glacier Basin, West Antarctica. Science.

[18] Rignot E (2019). Four decades of Antarctic ice sheet mass balance from 1979–2017. Proc. Natl Acad. Sci. USA.

[19] Shepherd A (2019). Trends in Antarctic ice sheet elevation and mass. Geophys. Res. Lett..

[20] Konrad H (2018). Net retreat of Antarctic glacier grounding lines. Nat. Geosci..

[21] Mouginot J, Rignot E, Scheuchl B (2014). Sustained increase in ice discharge from the Amundsen Sea Embayment, West Antarctica, from 1973 to 2013. Geophys. Res. Lett..

[22] Bjordal J, Storelvmo T, Alterskjær K, Carlsen T (2020). Equilibrium climate sensitivity above 5 °C plausible due to state-dependent cloud feedback. Nat. Geosci..

[23] Scheffer M, Hirota M, Holmgren M, Van Nes EH, Chapin FS (2012). Thresholds for boreal biome transitions. Proc. Natl Acad. Sci. USA.

[24] Abis B, Brovkin V (2017). Environmental conditions for alternative tree-cover states in high latitudes. Biogeosciences.

[25] Hirota M, Holmgren M, Van Nes EH, Scheffer M (2011). Global resilience of tropical forest and savanna to critical transitions. Science.

[26] Staver AC, Archibald S, Levin SA (2011). The global extent and determinants of Savanna and forest as alternative biome states. Science.

[27] Wuyts B, Champneys AR, House JI (2017). Amazonian forest-savanna bistability and human impact. Nat. Commun..

[28] Verbesselt J (2016). Remotely sensed resilience of tropical forests. Nat. Clim. Change.

[29] Boulton CA, Lenton TM, Boers N (2022). Pronounced loss of Amazon rainforest resilience since the early 2000s. Nat. Clim. Change.

[30] Lenton TM (2022). A resilience sensing system for the biosphere. Philos. Trans. R. Soc. B: Biol. Sci..

[31] Kéfi S, Dakos V, Scheffer M, Van Nes EH, Rietkerk M (2012). Early warning signals also precede non-catastrophic transitions. Oikos.

[32] Boers N, Ghil M, Stocker TF (2022). Theoretical and paleoclimatic evidence for abrupt transitions in the Earth system. Environ. Res. Lett..

[33] Robinson A, Calov R, Ganopolski A (2012). Multistability and critical thresholds of the Greenland ice sheet. Nat. Clim. Change.

[34] Liu, W., Xie, S.-P., Liu, Z. & Zhu, J. Overlooked possibility of a collapsed Atlantic Meridional Overturning Circulation in warming climate. *Sci. Adv.* **3**, e1601666 (2017). 10.1126/sciadv.1601666 PMC5217057 28070560

[35] Jackson LC, Wood RA (2018). Hysteresis and Resilience of the AMOC in an Eddy-Permitting GCM. Geophys. Res. Lett..

[36] Boers N, Rypdal M (2021). Critical slowing down suggests that the western Greenland ice sheet is close to a tipping point. Proc. Natl Acad. Sci. USA.

[37] Boers N (2021). Observation-based early-warning signals for a collapse of the Atlantic Meridional Overturning Circulation. Nat. Clim. Change.

[38] Moesinger L (2020). The global long-term microwave Vegetation Optical Depth Climate Archive (VODCA). Earth Syst. Sci. Data.

[39] Boulton CA, Allison LC, Lenton TM (2014). Early warning signals of Atlantic Meridional Overturning Circulation collapse in a fully coupled climate model. Nat. Commun..

[40] Ciemer C (2019). Higher resilience to climatic disturbances in tropical vegetation exposed to more variable rainfall. Nat. Geosci..

[41] Dakos V, van Nes E, Donangelo R, Fort H, Scheffer M (2010). Spatial correlation as leading indicator of catastrophic shifts. Theor. Ecol..

[42] Krishnamurthy R PK, Fisher JB, Schimel DS, Kareiva PM (2020). Applying tipping point theory to remote sensing science to improve early warning drought signals for food security. Earth’s Future.

[43] Krishnamurthy R PK, Fisher JB, Choularton RJ, Kareiva PM (2022). Anticipating drought-related food security changes. Nat. Sustain..

[44] Thellmann K (2018). Tipping points in the supply of ecosystem services of a mountainous watershed in Southeast Asia. Sustainability.

[45] Mercer JH (1978). West Antarctic ice sheet and CO_2_ greenhouse effect: a threat of disaster. Nature.

[46] Lenton TM (2019). Climate tipping points—too risky to bet against. Nature.

[47] Hall A, Cox P, Huntingford C, Klein S (2019). Progressing emergent constraints on future climate change. Nat. Clim. Change.

[48] Cai Y, Lenton TM, Lontzek TS (2016). Risk of multiple interacting tipping points should encourage rapid CO_2_ emission reduction. Nat. Clim. Change.

[49] Committee on Climate Change. *Net Zero—The UK’s Contribution to Stopping Global Warming*. (Committee on Climate Change, 2019).

[50] Lenton TM, Ciscar J-C (2013). Integrating tipping points into climate impact assessments. Clim. Change.

[51] Collins, M. et al. in *The Ocean and Cryosphere in a Changing Climate: Special Report of the Intergovernmental Panel on Climate Change* (eds H.-O. Pörtner et al.) 589–656 (Cambridge University Press, 2019).

[52] Sellers PJ, Schimel DS, Moore B, Liu J, Eldering A (2018). Observing carbon cycle-climate feedbacks from space. Proc. Natl Acad. Sci. USA.

[53] Berdugo M, Gaitán JJ, Delgado-Baquerizo M, Crowther TW, Dakos V (2022). Prevalence and drivers of abrupt vegetation shifts in global drylands. Proc. Natl Acad. Sci. USA.

[54] Smith T, Traxl D, Boers N (2022). Empirical evidence for recent global shifts in vegetation resilience. Nat. Clim. Change.

[55] Scheffer M, Carpenter S, Foley JA, Folke C, Walker B (2001). Catastrophic shifts in ecosystems. Nature.

[56] Biggs, R., Peterson, G. D. & Rocha, J. C. The Regime Shifts Database: a framework for analyzing regime shifts in social-ecological systems. *Ecol. Soc.* **23**, 9 (2018).

[57] Swingedouw D (2021). On the risk of abrupt changes in the North Atlantic subpolar gyre in CMIP6 models. Ann. N. Y. Acad. Sci..

[58] Swingedouw D (2020). Early warning from space for a few key tipping points in physical, biological, and social-ecological systems. Surv. Geophys..

[59] Kumar P, Kishtawal CM, Pal PK (2014). Impact of satellite rainfall assimilation on Weather Research and Forecasting model predictions over the Indian region. J. Geophys. Res.: Atmos..

[60] Pattyn F, Morlighem M (2020). The uncertain future of the Antarctic ice sheet. Science.

[61] Rosier SHR (2021). The tipping points and early warning indicators for Pine Island Glacier, West Antarctica. Cryosphere.

[62] Feldmann J, Levermann A (2015). Collapse of the West Antarctic ice sheet after local destabilization of the Amundsen Basin. Proc. Natl Acad. Sci..

[63] Staal A (2018). Forest-rainfall cascades buffer against drought across the Amazon. Nat. Clim. Change.

[64] Hansen MC (2013). High-Resolution Global Maps of 21st-Century Forest Cover Change. Science.

[65] Hansen MC (2020). The fate of tropical forest fragments. Sci. Adv..

[66] Inderjit, Callaway RM, Meron E (2021). Belowground feedbacks as drivers of spatial self-organization and community assembly. Phys. Life Rev..

[67] Rietkerk M (2021). Evasion of tipping in complex systems through spatial pattern formation. Science.

[68] Buxton JE (2021). Quantitatively monitoring the resilience of patterned vegetation in the Sahel. Glob. Change Biol..

[69] Druckenbrod DL (2019). Redefining temperate forest responses to climate and disturbance in the eastern United States: New insights at the mesoscale. Glob. Ecol. Biogeogr..

[70] Boulton C, Lenton T (2019). A new method for detecting abrupt shifts in time series. F1000Research.

[71] Bernardino PN (2020). Global-scale characterization of turning points in arid and semi-arid ecosystem functioning. Glob. Ecol. Biogeogr..

[72] Nitze I, Grosse G, Jones BM, Romanovsky VE, Boike J (2018). Remote sensing quantifies widespread abundance of permafrost region disturbances across the Arctic and Subarctic. Nat. Commun..

[73] Kumar SS (2019). Alternative vegetation states in tropical forests and Savannas: the search for consistent signals in diverse remote sensing data. Remote Sens..

[74] Zeng Y (2019). Towards a traceable climate service: assessment of quality and usability of essential climate variables. Remote Sens..

[75] Duveiller G (2021). Revealing the widespread potential of forests to increase low level cloud cover. Nat. Commun..

[76] Deng Z (2022). Comparing national greenhouse gas budgets reported in UNFCCC inventories against atmospheric inversions. Earth Syst. Sci. Data.

[77] Sorg A, Bolch T, Stoffel M, Solomina O, Beniston M (2012). Climate change impacts on glaciers and runoff in Tien Shan (Central Asia). Nat. Clim. Change.

[78] Watson SCL (2021). Does agricultural intensification cause tipping points in ecosystem services?. Landsc. Ecol..

[79] Miralles DG, Teuling AJ, van Heerwaarden CC, Vilà-Guerau de Arellano J (2014). Mega-heatwave temperatures due to combined soil desiccation and atmospheric heat accumulation. Nat. Geosci..

[80] Reichstein M (2007). Reduction of ecosystem productivity and respiration during the European summer 2003 climate anomaly: a joint flux tower, remote sensing and modelling analysis. Glob. Change Biol..

[81] Witte JC (2011). NASA A-Train and Terra observations of the 2010 Russian wildfires. Atmos. Chem. Phys..

[82] Shaposhnikov D (2014). Mortality related to air pollution with the Moscow heat wave and wildfire of 2010. Epidemiology.

[83] Hunt E (2021). Agricultural and food security impacts from the 2010 Russia flash drought. Weather Clim. Extremes.

[84] Kopp RE, Shwom RL, Wagner G, Yuan J (2016). Tipping elements and climate–economic shocks: Pathways toward integrated assessment. Earth’s Future.

[85] Milkoreit M (2022). Social tipping points everywhere?—Patterns and risks of overuse. WIREs Clim. Change.

[86] Mortimer C (2022). Benchmarking algorithm changes to the Snow CCI+ snow water equivalent product. Remote Sens. Environ..

[87] Paul S, Hendricks S, Ricker R, Kern S, Rinne E (2018). Empirical parametrization of Envisat freeboard retrieval of Arctic and Antarctic sea ice based on CryoSat-2: progress in the ESA Climate Change Initiative. Cryosphere.

[88] Wissel C (1984). A universal law of the characteristic return time near thresholds. Oecologia.

[89] De Keersmaecker W (2022). Evaluating recovery metrics derived from optical time series over tropical forest ecosystems. Remote Sens. Environ..

[90] Kubo R (1966). The fluctuation-dissipation theorem. Rep. Prog. Phys..

[91] De Keersmaecker W (2014). How to measure ecosystem stability? An evaluation of the reliability of stability metrics based on remote sensing time series across the major global ecosystems. Glob. Change Biol..

[92] Forzieri G, Dakos V, McDowell NG, Ramdane A, Cescatti A (2022). Emerging signals of declining forest resilience under climate change. Nature.

[93] De Keersmaecker W (2015). A model quantifying global vegetation resistance and resilience to short-term climate anomalies and their relationship with vegetation cover. Glob. Ecol. Biogeogr..

[94] Liu Y, Kumar M, Katul GG, Porporato A (2019). Reduced resilience as an early warning signal of forest mortality. Nat. Clim. Change.

[95] King MD (2020). Dynamic ice loss from the Greenland ice sheet driven by sustained glacier retreat. Commun. Earth Environ..

[96] Khan SA (2022). Accelerating ice loss from peripheral glaciers in North Greenland. Geophys. Res. Lett..

[97] Michel SLL (2022). Early warning signal for a tipping point suggested by a millennial Atlantic Multidecadal Variability reconstruction. Nat. Commun..

[98] Sgubin, G., Swingedouw, D., Drijfhout, S., Mary, Y. & Bennabi, A. Abrupt cooling over the North Atlantic in modern climate models. *Nat. Commun.* **8**, 14375 (2017). **Identifies a tipping point of deep convection collapse in the North Atlantic subpolar gyre occurring in several climate models at low levels of global warming**. 10.1038/ncomms14375 PMC5330854 28198383

[99] Knudby A, Jupiter S, Roelfsema C, Lyons M, Phinn S (2013). Mapping coral reef resilience indicators using field and remotely sensed data. Remote Sens..

[100] Tantet A, van der Burgt FR, Dijkstra HA (2015). An early warning indicator for atmospheric blocking events using transfer operators. Chaos: Interdiscip. J. Nonlinear Sci..

[101] Lenton TM (2011). Early warning of climate tipping points. Nat. Clim. Change.

[102] Turner MG (2020). Climate change, ecosystems and abrupt change: science priorities. Philos. Trans. R. Soc. B: Biol. Sci..

[103] Arani BMS, Carpenter SR, Lahti L, Nes EHV, Scheffer M (2021). Exit time as a measure of ecological resilience. Science.

[104] Hassanibesheli F, Boers N, Kurths J (2020). Reconstructing complex system dynamics from time series: a method comparison. N. J. Phys..

[105] Gilarranz LJ, Narwani A, Odermatt D, Siber R, Dakos V (2022). Regime shifts, trends, and variability of lake productivity at a global scale. Proc. Natl Acad. Sci. USA.

[106] van Nes EH, Scheffer M (2005). Implications of spatial heterogeneity for catastrophic regime shifts in ecosystems. Ecology.

[107] Bathiany S, Claussen M, Fraedrich K (2013). Detecting hotspots of atmosphere-vegetation interaction via slowing down—Part 1: a stochastic approach. Earth Syst. Dynam..

[108] Boers N, Marwan N, Barbosa HMJ, Kurths J (2017). A deforestation-induced tipping point for the South American monsoon system. Sci. Rep..

[109] Claussen M, Bathiany S, Brovkin V, Kleinen T (2013). Simulated climate–vegetation interaction in semi-arid regions affected by plant diversity. Nat. Geosci..

[110] Guttal V, Jayaprakash C (2009). Spatial variance and spatial skewness: leading indicators of regime shifts in spatial ecological systems. Theor. Ecol..

[111] Kéfi S (2014). Early warning signals of ecological transitions: methods for spatial patterns. PLoS ONE.

[112] Nijp JJ (2019). Spatial early warning signals for impending regime shifts: a practical framework for application in real-world landscapes. Glob. Change Biol..

[113] Eby S, Agrawal A, Majumder S, Dobson AP, Guttal V (2017). Alternative stable states and spatial indicators of critical slowing down along a spatial gradient in a savanna ecosystem. Glob. Ecol. Biogeogr..

[114] Majumder S, Tamma K, Ramaswamy S, Guttal V (2019). Inferring critical thresholds of ecosystem transitions from spatial data. Ecology.

[115] Hegerl, G. C. et al. Toward consistent observational constraints in climate predictions and projections. *Front. Clim.* **3**, 678109 (2021).

[116] Shiogama H, Watanabe M, Kim H, Hirota N (2022). Emergent constraints on future precipitation changes. Nature.

[117] Cox PM (2013). Sensitivity of tropical carbon to climate change constrained by carbon dioxide variability. Nature.

[118] Keenlyside NS, Latif M, Jungclaus J, Kornblueh L, Roeckner E (2008). Advancing decadal-scale climate prediction in the North Atlantic sector. Nature.

[119] Counillon F, Sakov P, Bertino L (2009). Application of a hybrid EnKF-OI to ocean forecasting. Ocean Sci..

[120] Smith DM (2020). North Atlantic climate far more predictable than models imply. Nature.

[121] Séférian R (2014). Multiyear predictability of tropical marine productivity. Proc. Natl Acad. Sci. USA.

[122] Li H, Ilyina T, Müller WA, Sienz F (2016). Decadal predictions of the North Atlantic CO2 uptake. Nat. Commun..

[123] Kriegler E, Hall JW, Held H, Dawson R, Schellnhuber HJ (2009). Imprecise probability assessment of tipping points in the climate system. Proc. Natl Acad. Sci. USA.

[124] Bakker P (2016). Fate of the Atlantic meridional overturning circulation: strong decline under continued warming and Greenland melting. Geophys. Res. Lett..

[125] Madsen MS (2022). The role of an interactive Greenland ice sheet in the coupled climate-ice sheet model EC-Earth-PISM. Clim. Dyn..

[126] Stouffer RJ (2006). Investigating the causes of the response of the thermohaline circulation to past and future climate changes. J. Clim..

[127] Swingedouw D (2013). Decadal fingerprints of freshwater discharge around Greenland in a multi-model ensemble. Clim. Dyn..

[128] Swingedouw, D. et al. AMOC Recent and Future Trends: A crucial role for oceanic resolution and Greenland melting? *Front. Clim.* **4**, (2022). **Shows that Greenland ice sheet melt can significantly weaken deep convection in the North Atlantic subpolar gyre, but this is not captured in the latest coupled climate models**.

[129] Mosblech NAS (2012). North Atlantic forcing of Amazonian precipitation during the last ice age. Nat. Geosci..

[130] Jomelli V (2022). In-phase millennial-scale glacier changes in the tropics and North Atlantic regions during the Holocene. Nat. Commun..

[131] Ciemer C, Winkelmann R, Kurths J, Boers N (2021). Impact of an AMOC weakening on the stability of the southern Amazon rainforest. Eur. Phys. J. Spec. Top..

[132] Good P, Boers N, Boulton CA, Lowe JA, Richter I (2022). How might a collapse in the Atlantic Meridional Overturning Circulation affect rainfall over tropical South America?. Clim. Resil. Sustain..

[133] Runge J (2018). Causal network reconstruction from time series: From theoretical assumptions to practical estimation. Chaos: Interdiscip. J. Nonlinear Sci..

[134] Runge J (2019). Inferring causation from time series in Earth system sciences. Nat. Commun..

[135] Reich BJ (2021). A review of spatial causal inference methods for environmental and epidemiological applications. Int. Stat. Rev..

[136] Pérez-Suay A, Camps-Valls G (2019). Causal inference in geoscience and remote sensing from observational data. IEEE Trans. Geosci. Remote Sens..

[137] Kretschmer M, Coumou D, Donges JF, Runge J (2016). Using causal effect networks to analyze different Arctic drivers of midlatitude winter circulation. J. Clim..

[138] Papagiannopoulou C (2017). A non-linear Granger-causality framework to investigate climate–vegetation dynamics. Geosci. Model Dev..

[139] Reygadas Y, Jensen JLR, Moisen GG, Currit N, Chow ET (2020). Assessing the relationship between vegetation greenness and surface temperature through Granger causality and Impulse-Response coefficients: a case study in Mexico. Int. J. Remote Sens..

[140] Sugihara G (2012). Detecting causality in complex ecosystems. Science.

[141] Smith T (2023). Reliability of resilience estimation based on multi-instrument time series. Earth Syst. Dyn..

[142] Bury TM (2021). Deep learning for early warning signals of regime shifts. Proc. Natl Acad. Sci. USA.

[143] Dylewsky D (2023). Universal early warning signals of phase transitions in climate systems. J. R. Soc. Interface.

[144] Bathiany S, Hidding J, Scheffer M (2020). Edge detection reveals abrupt and extreme climate events. J. Clim..

[145] Popp T (2020). Consistency of satellite climate data records for Earth system monitoring. Bull. Am. Meteorol. Soc..

[146] Plummer S, Lecomte P, Doherty M (2017). The ESA Climate Change Initiative (CCI): a European contribution to the generation of the Global Climate Observing System. Remote Sens. Environ..

[147] White HJ (2020). Quantifying large-scale ecosystem stability with remote sensing data. Remote Sens. Ecol. Conserv..

[148] Bousquet E (2021). Influence of surface water variations on VOD and biomass estimates from passive microwave sensors. Remote Sens. Environ..

[149] Tao S (2022). Increasing and widespread vulnerability of intact tropical rainforests to repeated droughts. Proc. Natl Acad. Sci. USA.

[150] Beaugrand G (2019). Prediction of unprecedented biological shifts in the global ocean. Nat. Clim. Change.

[151] Green, H. L., Findlay, H. S., Shutler, J. D., Land, P. E. & Bellerby, R. G. J. Satellite Observations Are Needed to Understand Ocean Acidification and Multi-Stressor Impacts on Fish Stocks in a Changing Arctic Ocean. *Front. Marine Sci.* **8**, 635797 (2021).

[152] Melet A (2020). Earth observations for monitoring marine coastal hazards and their drivers. Surv. Geophys..

[153] Foo, S. A. & Asner, G. P. Scaling up coral reef restoration using remote sensing technology. *Front. Marine Sci.* **6**, 79 (2019).

[154] Staal A, Dekker SC, Hirota M, van Nes EH (2015). Synergistic effects of drought and deforestation on the resilience of the south-eastern Amazon rainforest. Ecol. Complex..

[155] Zemp DC (2017). Self-amplified Amazon forest loss due to vegetation-atmosphere feedbacks. Nat. Commun..

[156] van Belzen J (2017). Vegetation recovery in tidal marshes reveals critical slowing down under increased inundation. Nat. Commun..

[157] Alibakhshi S, Groen T, Rautiainen M, Naimi B (2017). Remotely-sensed early warning signals of a critical transition in a wetland ecosystem. Remote Sens..

[158] Tehrani, N. A. & Janalipour, M. Predicting ecosystem shift in a Salt Lake by using remote sensing indicators and spatial statistics methods (case study: Lake Urmia basin). *Environ. Eng. Res*. **26**, 200225–200220 (2021).

[159] Lees KJ (2021). Using remote sensing to assess peatland resilience by estimating soil surface moisture and drought recovery. Sci. Total Environ..

[160] Lees KJ, Buxton J, Boulton CA, Abrams JF, Lenton TM (2021). Using satellite data to assess management frequency and rate of regeneration on heather moorlands in England as a resilience indicator. Environ. Res. Commun..

[161] Miner KR (2022). Permafrost carbon emissions in a changing Arctic. Nat. Rev. Earth Environ..

[162] Talib J (2022). The sensitivity of the West African monsoon circulation to intraseasonal soil moisture feedbacks. Q. J. R. Meteorol. Soc..

[163] IPCC. *Global Warming of 1.5* *°C. An IPCC Special Report on the Impacts of Global Warming of 1.5* *°C above Pre-industrial Levels and Related Global Greenhouse Gas Emission Pathways, in the Context of Strengthening the Global Response to the Threat of Climate Change, Sustainable Development, and Efforts to Eradicate Poverty*. (IPCC, 2018).

[164] IPCC. *IPCC Special Report on the Ocean and Cryosphere in a Changing Climate*. (IPCC, 2019). 10.1007/s13280-019-01313-8 PMC7413947 31994026

[165] IPCC. *Climate Change 2021: The Physical Science Basis. Contribution of Working Group I to the Sixth Assessment Report of the Intergovernmental Panel on Climate Change*. (Cambridge University Press, 2021).

[166] Boulton CA, Lenton TM (2015). Slowing down of North Pacific climate variability and its implications for abrupt ecosystem change. Proc. Natl Acad. Sci. USA.

