# Publisher Correction: Remotely sensing potential climate change tipping points across scales

**DOI:** 10.1038/s41467-024-45881-0

**Published:** 2024-03-01

**Authors:** Timothy M. Lenton, Jesse F. Abrams, Annett Bartsch, Sebastian Bathiany, Chris A. Boulton, Joshua E. Buxton, Alessandra Conversi, Andrew M. Cunliffe, Sophie Hebden, Thomas Lavergne, Benjamin Poulter, Andrew Shepherd, Taylor Smith, Didier Swingedouw, Ricarda Winkelmann, Niklas Boers

**Affiliations:** 1 Global Systems Institute, University of Exeter, Exeter, UK; 2 b.geos GmbH, Industriestrasse 1A, 2100 Korneuburg, Austria; 3 Austrian Polar Research Institute, Vienna, Austria; 4 Earth System Modelling, School of Engineering & Design, Technical University of Munich, Munich, Germany; 5 Potsdam Institute for Climate Impact Research, Potsdam, Germany; 6 National Research Council of Italy, ISMAR-Lerici, Forte Santa Teresa, Loc. Pozzuolo, 19032 Lerici (SP), Italy; 7 Future Earth Secretariat, Stockholm, Sweden; 8 European Space Agency, ECSAT, Harwell, Oxfordshire, UK; 9 Norwegian Meteorological Institute, Oslo, Norway; 10 NASA Goddard Space Flight Centre, Greenbelt, MD 20771 USA; 11 Department of Geography and Environmental Sciences, Northumbria University, Newcastle, UK; 12 Institute of Geosciences, University of Potsdam, Potsdam, Germany; 13 University of Bordeaux, CNRS, Bordeaux INP, EPOC, UMR 5805, 33600 Pessac, France

**Keywords:** Climate-change impacts, Projection and prediction

Correction to: *Nature Communications* 10.1038/s41467-023-44609-w, published online 06 January 2024

The original version of this Article contained an error in Table 1, in which some cell boundaries were drawn incorrectly. The correct version of Table 1 contains the correct boundaries and capitalizes a few terms, while it also corrects the text in one cell from “elevation change” to “Surface elevation”. This has been corrected in both the PDF and HTML versions of the Article.

